# A Machine Learning and Wearable Sensor Based Approach to Estimate External Knee Flexion and Adduction Moments During Various Locomotion Tasks

**DOI:** 10.3389/fbioe.2020.00009

**Published:** 2020-01-24

**Authors:** Bernd J. Stetter, Frieder C. Krafft, Steffen Ringhof, Thorsten Stein, Stefan Sell

**Affiliations:** ^1^Institute of Sports and Sports Science, Karlsruhe Institute of Technology, Karlsruhe, Germany; ^2^Department of Sport and Sport Science, University of Freiburg, Freiburg, Germany; ^3^Joint Center Black Forest, Hospital Neuenbuerg, Neuenbuerg, Germany

**Keywords:** knee osteoarthritis, biomechanics, knee joint loading, biofeedback, artificial neural networks, accelerometers and gyroscopes, reduced sensor set

## Abstract

Joint moment measurements represent an objective biomechanical parameter of knee joint load in knee osteoarthritis (KOA). Wearable sensors in combination with machine learning techniques may provide solutions to develop assistive devices in KOA patients to improve disease treatment and to minimize risk of non-functional overreaching (e.g., pain). The purpose of this study was to develop an artificial neural network (ANN) that estimates external knee flexion moments (KFM) and external knee adduction moments (KAM) during various locomotion tasks, based on data obtained by two wearable sensors. Thirteen participants were instrumented with two inertial measurement units (IMUs) located on the right thigh and shank. Participants performed six different locomotion tasks consisting of linear motions and motions with a change of direction, while IMU signals as well as full body kinematics and ground reaction forces were synchronously recorded. KFM and KAM were determined using a full body biomechanical model. An ANN was trained to estimate the KFM and KAM time series using the IMU signals as input. Evaluation of the ANN was done using a leave-one-subject-out cross-validation. Concordance of the ANN-estimated KFM and reference data was categorized for five tasks (walking straight, 90° walking turn, moderate running, 90° running turn and 45° cutting maneuver) as strong (*r* ≥ 0.69, rRMSE ≤ 23.1) and as moderate for fast running (*r* = 0.65 ± 0.43, rRMSE = 25.5 ± 7.0%). For all locomotion tasks, KAM yielded a lower concordance in comparison to the KFM, ranging from weak (*r* ≤ 0.21, rRMSE ≥ 33.8%) in cutting and fast running to strong (*r* = 0.71 ± 0.26, rRMSE = 22.3 ± 8.3%) for walking straight. Smallest mean difference of classical discrete load metrics was seen for KFM impulse, 10.6 ± 47.0%. The results demonstrate the feasibility of using only two IMUs to estimate KFM and KAM to a limited extent. This methodological step facilitates further work that should aim to improve the estimation accuracy to provide valuable biofeedback systems for KOA patients. Greater accuracy of effective implementation could be achieved by a participant- or task-specific ANN modeling.

## Introduction

Medio-tibiofemoral knee osteoarthritis (KOA) is a major cause of disability in elderly people (Hurley et al., 1997) and accounts for high socio-economic burden in industrial countries (Neogi et al., 2009; Reeves and Bowling, 2011; Ferreira et al., 2015). Symptoms known as knee pain, functional impairment and a loss of mobility, can lead to physical and psychological disability and reduced quality of life (Bennell et al., 2011; Richards et al., 2017).

Mechanical factors, particularly the knee joint load have shown to profoundly influence the severity and progression of KOA (Sharma et al., 1998; Andriacchi and Muendermann, 2006; Foroughi et al., 2009; Bennell et al., 2011; Reeves and Bowling, 2011). A widely used surrogate measure of the compressive load of the medial compartment is the external knee adduction moment (KAM) (Sharma et al., 1998; Bennell et al., 2011; Reeves and Bowling, 2011; Ferreira et al., 2015). Moreover, the knee flexion moment (KFM) has been highlighted as a critical measure to assess the loading of the medial compartment (Walter et al., 2010; Ferreira et al., 2015; Cheung et al., 2018) as well as to quantify the progression of patellofemoral cartilage damage (Teng et al., 2015; Crossley et al., 2016).

Beside other non-pharmacological conservative treatments (e.g., bracing or footwear interventions) (Sarzi-Puttini et al., 2005; Reeves and Bowling, 2011), gait modification approaches by gait retraining therapies (e.g., modifying the foot progression angle) have shown to be effective to reduce the KAM during walking and to improve the symptoms of patients (Barrios et al., 2010; Cheung et al., 2018; Karatsidis et al., 2018). Richards et al. (2017) stated in their systematic review that a strong potential exists for the development of biofeedback systems for reducing KAM and pain and for improving knee joint function in KOA patients. The development of assistive devices (e.g., a smart knee sleeve to monitor the knee load in combination with a smartphone-based user feedback system) could help to provide effective disease-enhancing interventions to slow down the loss of cartilage volume (Shull et al., 2014). Additionally, as exercise is a key component of the KOA management (Bennell et al., 2011; Ferreira et al., 2015; Richards et al., 2017), assistive devices could be beneficial in supporting therapeutical exercise. However, most of the current studies with respect to the assessment of knee joint loading were conducted in a laboratory setting using motion capture and force plate measurements (Barrios et al., 2010; Richards et al., 2017; Cheung et al., 2018). The major shortcoming of such laboratory-based methods is that they cannot be completely included into a patients' habitual environment (Muro-de-la-Herran et al., 2014; Shull et al., 2014).

As a consequence, alternative measurement technologies have been provided progressive advances over the past years (Muro-de-la-Herran et al., 2014; Wong et al., 2015). One of the first studies toward a wearable measurement tool was done by van den Noort et al. (2011). The authors tested the effect of an instrumented force shoe in combination with an optoelectronic marker system on target variables (e.g., KAM) in 20 KOA patients. Therein, the authors stated the necessity of additional measurement equipment (e.g., inertial sensors) to obtain joint positions and orientations as a complement to ground reaction force (GRF) measurements in order to calculate the KAM. Karatsidis et al. (2016) compared GRF estimation accuracies of a full-body inertial motion capture and optical motion capture system due to the importance of the GRF measures as input in biomechanical analysis to estimate joint kinetics. Their results showed comparable results between the two systems. Therefore, the authors concluded that the inertial sensor-based system has a high potential in monitoring critical biomechanical parameters in habitual conditions. Yang and Mao (2015) postulated a method for evaluating the intersegmental forces and moments acting on the lower limbs during walking solely based on posture data obtained from seven inertial sensors placed on the lower limbs and trunk in combination with a 3D analytical model. In 2018 Karatsidis et al. proposed and evaluated a wearable visual feedback system for gait retraining using inertial sensing with seven inertial measurement units (IMUs) and augmented reality technologies. The foot progression angle was used for visual feedback and was tracked by the wearable system with a root mean square error of 2.4°, compared to an optical motion capture system. Knee joint kinetics were not analyzed in this study. A further approach of a mobile assessment of knee joint biomechanics in natural environment was recently provided by Konrath et al. (2019). The authors estimated the KAM and the tibio-femoral joint contact force during activities of daily living by means of combining musculoskeletal modeling with inertial motion capture (17 IMUs). The results showed comparable estimation accuracies for the IMU-based approach compared to the same musculoskeletal model using optical motion capture and force plate measurements.

The majority of applied methods require modeling of the musculoskeletal system to a certain degree, with mandatory embedded subject-specific anthropometric data (e.g., mass, dimensions, and center of mass of the body segments). However, such modeling processes inevitably introduce inaccuracies (van den Noort et al., 2013; Faber et al., 2016; Ancillao et al., 2018). In contrast, machine learning-based approaches do not need an *a priori* knowledge of the model as they build up their model by using training data (Sivakumar et al., 2016; Ancillao et al., 2018; Halilaj et al., 2018). Accurate predictions for new data can be made by learning the relationship between a set of independent variables (e.g., IMU signals) and one or more dependent variables (e.g., KAM) (Lin et al., 2016; Halilaj et al., 2018). Several studies have shown that machine learning techniques, such as artificial neural networks (ANN), are powerful tools to deduce biomechanical variables based on measured accelerations or angular velocities of body segments (Leporace et al., 2015; Guo et al., 2017; Ancillao et al., 2018; Wouda et al., 2018; Stetter et al., 2019). The study by Wouda et al. (2018) used an ANN approach to estimate vertical GRFs and sagittal knee kinematics during running, based on three inertial sensors placed at the lower legs and the pelvis. The estimated force-time profiles and flexion/extension profiles showed high agreement with the optical and GRF reference measure. In a recent study we presented an ANN approach to estimate knee joint forces in sport movements (Stetter et al., 2019). Good agreement between ANN-estimated outcomes and inverse dynamics-calculated vertical and anterior-posterior knee joint forces were shown, which highlights the feasibility of an ANN approach to estimate internal loadings on the knee joint structures.

Although the above described studies have estimated joint kinematics and kinetics during locomotion, no study has directly estimated biomechanical surrogate measures for knee joint load in KOA using an ambulatory minimal body-worn sensor setup so far. Therefore, the purpose of this study was to develop an ANN that estimates KFM and KAM during various locomotion tasks based on data obtained by two wearable sensors integrated in a knee sleeve. The findings of this study could help to (1) overcome current restrictions in the mobile assessment of knee joint loading in KOA patients and (2) open new possibilities in diagnosing the patients' habitual life, which could help to improve disease treatment strategies and minimizing the risk of non-functional overreaching (e.g., pain).

## Materials and Methods

### Participants

The current study used data from the sample presented in Stetter et al. (2019) and forms a secondary dataset analysis. The sample consisted of 13 healthy males (age, 26.1 ± 2.9 years; height, 178.7 ± 5.5 cm; body mass, 78.4 ± 5.9 kg). All participants exhibited bowlegs (minimum inter-knee distance of 0.05 m), which mimics the common varus malalignment of medial KOA patients (Bennell et al., 2011). All participants gave written informed consent in accordance with the Declaration of Helsinki. The study was approved by the ethics committee of the Karlsruhe Institute of Technology.

### Experimental Protocol

**Figure 1:**
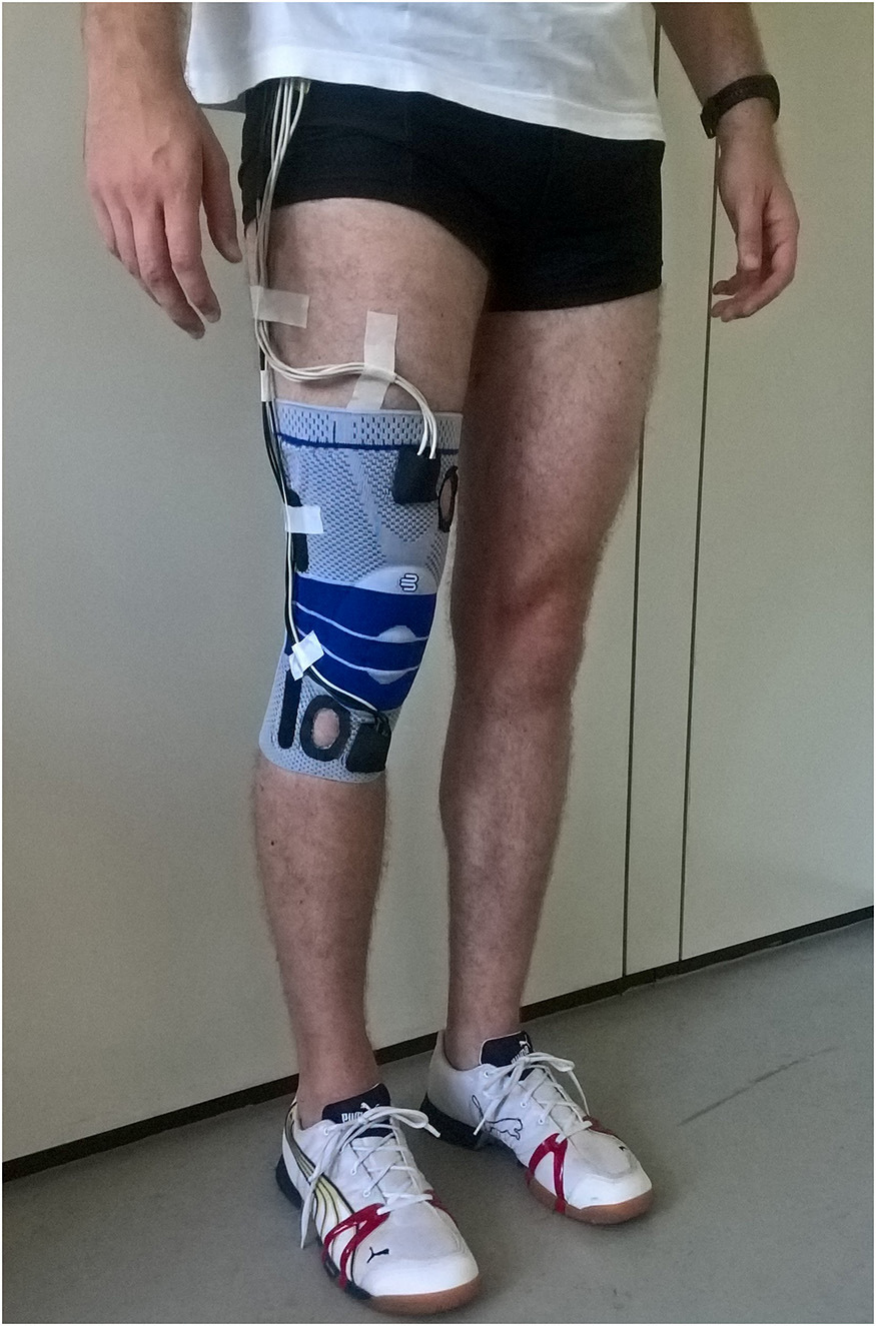
A participant wearing the knee sleeve on the right leg. The two inertial measurement units were placed in the patch pockets at the upper and lower frontal end of the knee sleeve.

Measurements were performed at the BioMotion Center, Institute of Sports and Sports Science, Karlsruhe Institute of Technology, Karlsruhe, Germany. Two identical custom-built 6DOF IMUs (1,500 Hz, ±8 g accelerometer, ±2,000°/s gyroscope) were attached to each participant's right leg while they performed six different locomotion tasks at self-selected speed: walking straight, 90° walking turn, moderate running, fast running, 90° running turn and 45° cutting maneuver. Participants were instructed to perform the 90° turns in clockwise direction. A detailed description of the right orientated cutting maneuver (named as v-cut) is described by Neptune et al. (1999). Participants were instructed to perform at least three successful trials of each task. A trial was considered successful when the right foot landed cleanly within the boundaries of a force plate. The IMUs were positioned in two patch pockets at the upper and lower frontal end of a customized knee sleeve (Figure 1). This positioning was chosen in order to capture IMU signals closely related to knee kinematics and dynamics, as the recent study by Matijevich et al. (2019) has highlighted that a targeted approach is necessary to obtain structure-specific loading.

Full body kinematics and GRFs (1,000 Hz, AMTI Inc., Watertown, MA) were collected synchronously using a marker-based motion capture system (11 MX-13 cameras, 200 Hz, Vicon, Oxford, UK) in order to perform biomechanical modeling. A total of 42 spherical reflective markers were placed on the participants' body segments in accordance to the ALASKA Dynamicus protocol (ALASKA, INSYS GmbH, Germany) (Härtel and Hermsdorf, 2006; Willwacher et al., 2017). Prior to the attachment of the data collection equipment, standardized anthropometric measurements were exhibited. The measurements consisted of a total of 22 length, width and circumference measures of the body segments. Prior to performing trials, a static calibration trial was recorded for each participant in a natural upright posture.

### Biomechanical Model

The 3D marker coordinates and GRF data were reconstructed and filtered with a 15 Hz low-pass filter (zero-phase Butterworth 4th order) (Kristianslund et al., 2012). Inverse dynamics modeling was performed using the full-body Dynamicus 9 model (Härtel and Hermsdorf, 2006; Willwacher et al., 2017). Each participant was individually scaled to the generic linked-segment model using the measured anthropometrics and the static calibration trial (Whittlesey and Robertson, 2014). In a next step, the marker trajectories and GRFs acquired from the dynamic trials were used to determine the knee flexion moment (KFM) and the knee adduction moment (KAM). A 20 N threshold of the vertical GRF was used to extract the stance phase for each locomotion movement (Milner and Paquette, 2015). KFM and KAM time series were time-normalized to 100 time steps representing 0–100% of the stance phase. Joint moment amplitudes were normalized to body weight and expressed as external moments.

### Machine Learning Model

ANN modeling was set up with the Neural Network Toolbox in MATLAB R2019a (The MathWorks, USA). The IMU signals were low-pass filtered (zero-phase Butterworth 4th order filter; cut-off frequency of 15 Hz) and each trial was cropped to contain data for the same phase as the biomechanical data. An IMU signal matrix (rows: 13 participants × three trials × six tasks × 100 time steps; columns: two locations × six spatial dimensions) and a biomechanical data matrix (rows: 13 participants × three trials × six tasks × 100 time steps; columns: two variables) were created by vertically concatenating the IMU signals and KFM and KAM time series of all trials, respectively. An ANN was trained to model the association between the IMU signals and the KFM and KAM time series. The IMU signal matrix served as input and the biomechanical data matrix served as output (target). As a consequence, the ANN had 12 and two variables (i.e., nodes) in its input and output layer, respectively. The ANN architecture was inspired by previous work (Favre et al., 2012; Wouda et al., 2018) and had two hidden layers with 100 and 20 neurons, which were connected to the input and output nodes. The hidden layers and the output layer consisted of hyperbolic tangent sigmoid transfer functions and a linear transfer function, respectively. Initialization of the ANN was done using the Nguyen-Widrow initialization function. The ANN was trained for 1,000 iterations with Levenberg-Marquardt back-propagated error correction (Watson and Moré, 1978) and training was stopped if the gradient did not decrease for six consecutive epochs or if the gradient was smaller than 1 × 10^−6^. Evaluation of the ANN was done using a leave-one-subject-out cross-validation (Halilaj et al., 2018). The cross-validation involved training the ANN with all trials from 12 participants (i.e., the training set) and then testing with the trials from the remaining participant (i.e., the test set). As cross-dependencies between the input and output in a combined estimation model for KFM and KAM may affect the estimation accuracy (Wouda et al., 2018), independent models for KFM and KAM were also build. Independent models were trained and evaluated in the same manner as the combined model, beside the fact that only one variable was chosen in its output layer.

### Statistical Analysis

According to previous studies, for each movement, the agreement between the ANN-estimated outcomes (KFM^*^ and KAM^*^) and the inverse dynamics-calculated data (KFM and KAM) was derived from Pearson's correlation coefficients, which were categorized as weak (*r* ≤ 0.35), moderate (0.35 < *r* ≤ 0.67), strong (0.67 < *r* ≤ 0.90) and excellent (*r* > 0.90) (Taylor, 1990; Fluit et al., 2014; Karatsidis et al., 2016). Additionally, the Root Mean Squared Error (RMSE) and relative Root Mean Squared Error (rRMSE) were determined to assess the accuracy of the ANN estimations (Ren et al., 2008). The rRMSE facilitates the comparison between the different locomotion tasks with different moment amplitudes. The averages and standard deviations were calculated for *r*, RMSE and rRMSE from the 13 cross-validation subsets. Average *r* values across participants were computed using Fisher's z transformation (Corey et al., 1998). Mean *r* values were expressed in the original range from −1 to 1 by reversing the transformation. Furthermore, peak KFM^*^ and KFM^*^ impulse as well as peak KAM and KAM impulse were evaluated as classical discrete load metrics (Bennell et al., 2011; Teng et al., 2015). Impulse represents the area under the corresponding moment-time curve. Percent differences (%Diff) between ANN-estimated and inverse dynamics-calculated peak and impulse metrics were used to provide a pragmatic interpretation.

## Results

### Estimated Continuous Outcomes

**Figure 2:**
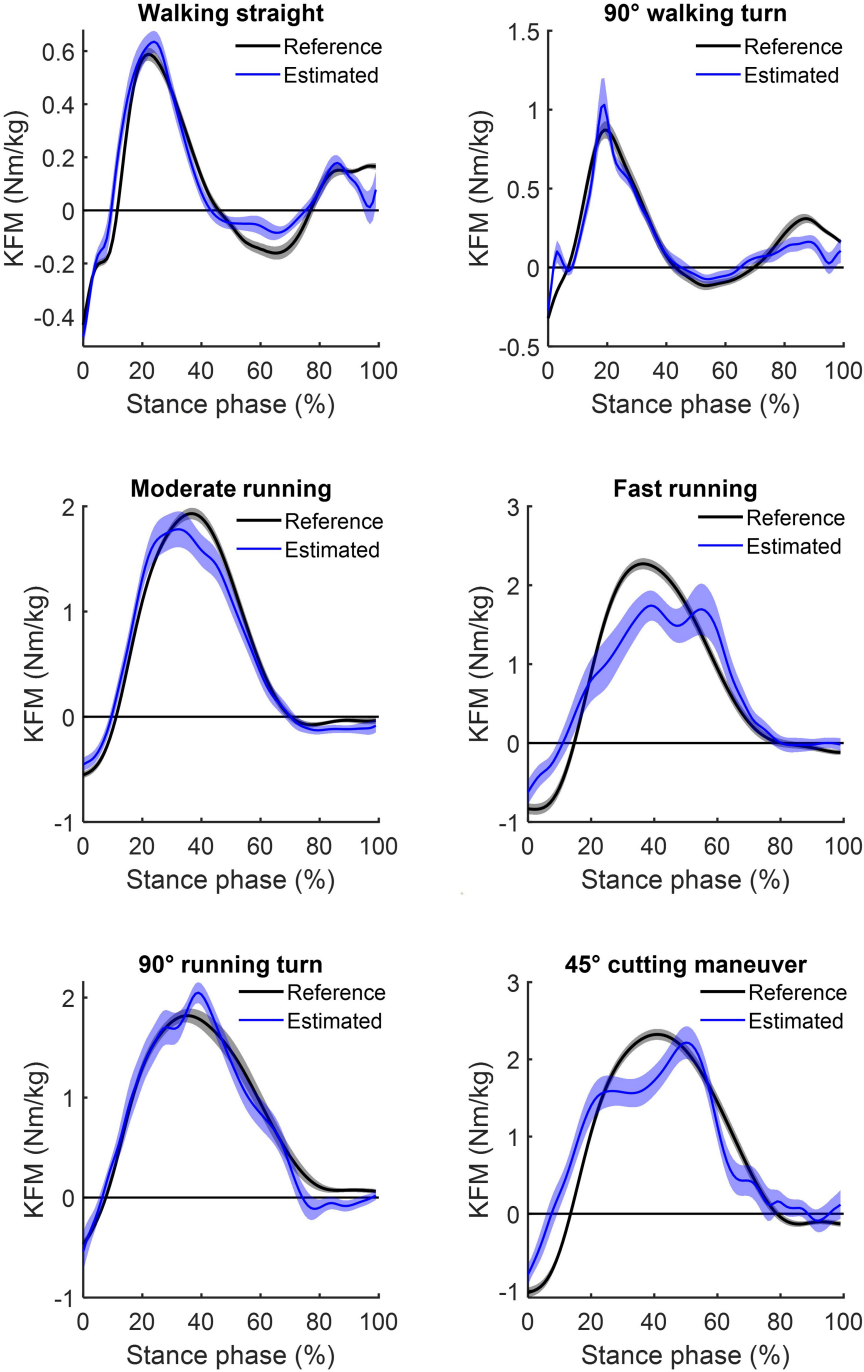
Mean (and standard error) of the estimated knee flexion moments (blue) for the six analyzed locomotion tasks compared to their respective inverse dynamics-calculated values (black). Positive values indicate external flexion moments and negative values indicate external extension moments.

**Figure 3:**
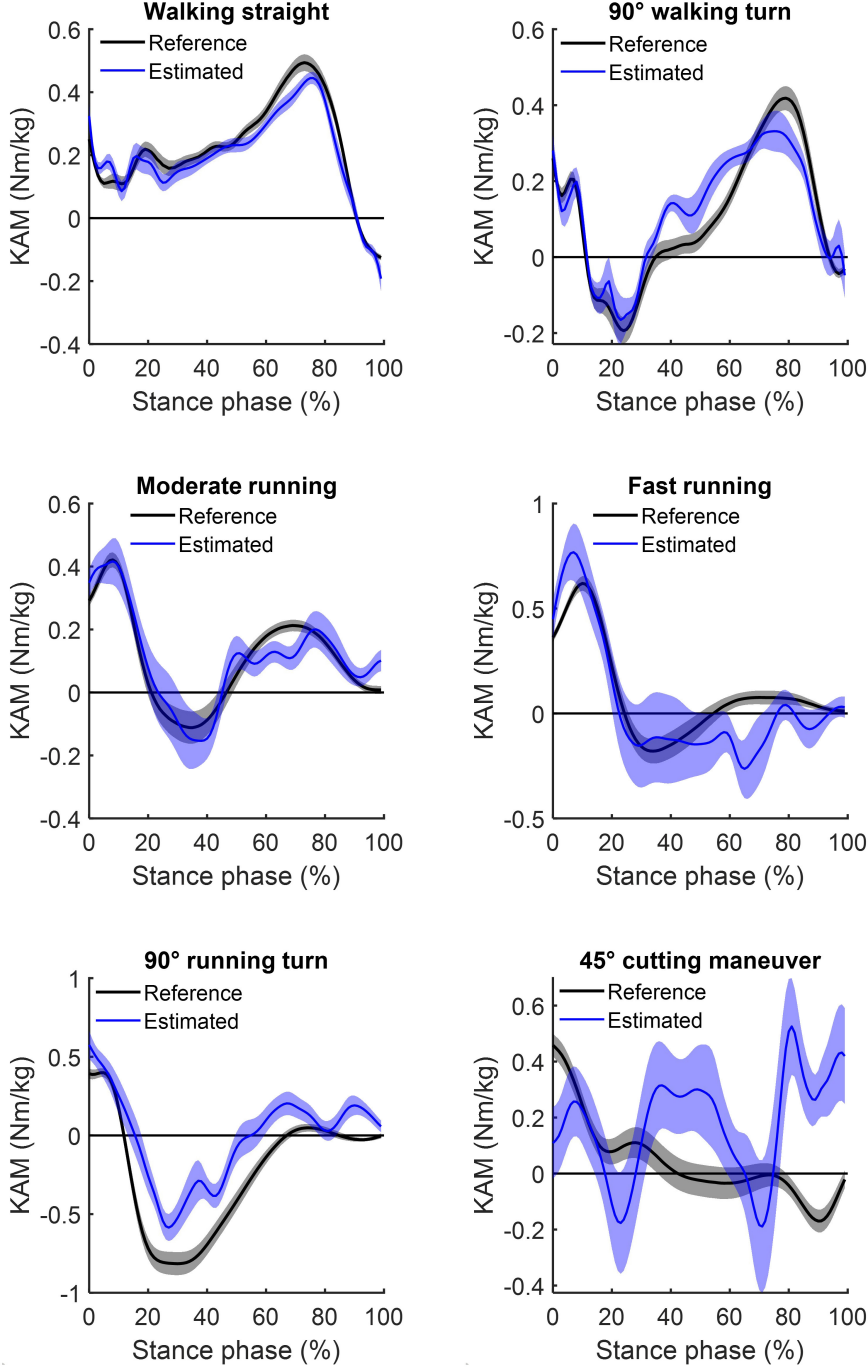
Mean (and standard error) of the estimated knee adduction moments (blue) for the six analyzed locomotion tasks compared to their respective inverse dynamics-calculated values (black). Positive values indicate external adduction moments and negative values indicate external abduction moments.

The ANN-estimated KFM^*^ and KAM^*^ time series of the whole stance phase are illustrated in Figures 2, 3, respectively, with the measured references used for comparison. An overview of the estimated accuracy for all movements is presented in Table 1.

**Table 1:** Accuracy (*r*, Pearson's correlation coefficient; RMSE, root-mean-squared error; rRMSE, relative root-mean-squared error) of the estimated continuous outcomes [knee flexion moment (KFM^*^), and knee adduction moment (KAM^*^)].

**Locomotion task**	KFM^*^			KAM^*^		
	***r***	**RMSE (Nm/kg)**	**rRMSE (%)**	***r***	**RMSE (Nm/kg)**	**rRMSE (%)**
Walking straight	0.72 ± 0.32	0.26 ± 0.09	18.4 ± 5.3	0.71 ± 0.26	0.18 ± 0.06	22.3 ± 8.3
90° walking turn	0.69 ± 0.31	0.32 ± 0.10	17.2 ± 3.1	0.56 ± 0.33	0.29 ± 0.10	23.9 ± 6.4
Moderate running	0.85 ± 0.43	0.58 ± 0.20	19.7 ± 7.9	0.40 ± 0.35	0.37 ± 0.14	34.4 ± 13.5
Fast running	0.65 ± 0.43	1.13 ± 0.46	25.5 ± 7.0	0.21 ± 0.47	0.80 ± 0.46	33.8 ± 8.5
90° running turn	0.79 ± 0.28	0.77 ± 0.20	20.8 ± 4.5	0.51 ± 0.22	0.62 ± 0.19	27.9 ± 3.9
45° cutting maneuver	0.73 ± 0.41	1.05 ± 0.41	23.1 ± 6.5	−0.05 ± 0.30	0.92 ± 0.54	37.2 ± 7.8
Mean	0.74 ± 0.36	0.67 ± 0.24	20.8 ± 5.7	0.39 ± 0.32	0.53 ± 0.25	29.9 ± 8.1

*Data are presented as mean ± standard deviations. Mean r and r standard deviation were computed using Fisher's z transformation*.

For the different locomotion tasks, the ANN-estimated time series revealed moderate to strong correlations for the KFM^*^ and weak to strong correlations for the KAM^*^. The highest correlation for KFM^*^ and KAM^*^ was observed for moderate running (*r* = 0.85 ± 0.43; mean ± standard deviation) and for walking straight (0.71 ± 0.26), respectively. For all locomotion tasks, the RMSE for KFM^*^ was between 0.26 ± 0.09 and 1.13 ± 0.46 Nm/kg, whereas for KAM^*^, that was between 0.18 ± 0.06 and 0.92 ± 0.54 Nm/kg. The rRMSE for the different locomotion tasks ranged between 17.2 ± 3.1% (walking 90° turn) and 25.5 ± 7.0% (fast running) for KFM^*^ and between 22.3 ± 8.3% (walking straight) and 37.2 ± 7.8% (cutting maneuver) for KAM^*^.

### Discrete Load Metrics

The inverse dynamics-calculated and ANN-estimated discrete load metrics (peak moments and moment integrals) are shown in Table 2. Table 3 presents the %Diff results for each of the performed locomotion tasks. The 90° walking turn showed the smallest %Diff (6.7 ± 31.3%) for the ANN-estimated KFM impulse in comparison to the reference values. In contrast, %Diff of KAM impulse were higher with a minimum value of 42.7 ± 108.9% for moderate running. The smallest %Diff for the estimation of peak KFM and KAM was 24.7 ± 33.0% (moderate running) and 39.1 ± 101.0% (walking straight), respectively. Across all locomotion tasks, mean differences of peak moments and moment integrals were lower for the KFM^*^ in comparison to the KAM^*^ (40.4 ± 56.5 vs. 130.3 ± 157.3% and 10.6 ± 47.0 vs. 161.4 ± 252.8%, respectively).

**Table 2:** Inverse dynamics-calculated (KFM and KAM) and ANN-estimated (KFM^*^ and KAM^*^) discrete load metrics (peak and impulse).

**Locomotion task**	**KFM**		**KAM**		KFM^*^		KAM^*^	
	**Peak**	**Impulse**	**Peak**	**Impulse**	**Peak**	**Impulse**	**Peak**	**Impulse**
	**(Nm/kg)**	**(Nm/kg)**	**(Nm/kg)**	**(Nm/kg)**	**(Nm/kg)**	**(Nm/kg)**	**(Nm/kg)**	**(Nm/kg)**
Walking straight	0.67 ± 0.13	45.72 ± 14.52	0.54 ± 0.15	69.16 ± 26.03	0.91 ± 0.30	52.31 ± 24.83	0.65 ± 0.18	64.23 ± 13.76
90° walking turn	1.02 ± 0.38	71.79 ± 36.05	0.57 ± 0.18	44.65 ± 21.96	1.55 ± 1.19	70.12 ± 31.23	0.90 ± 0.44	52.06 ± 17.00
Moderate running	2.03 ± 0.34	193.05 ± 58.08	0.52 ± 0.16	43.48 ± 21.81	2.57 ± 0.92	197.00 ± 90.16	0.84 ± 0.39	56.35 ± 50.26
Fast running	2.49 ± 0.35	246.20 ± 71.51	0.77 ± 0.20	51.35 ± 27.01	3.44 ± 1.92	259.80 ± 118.59	1.72 ± 0.99	91.98 ± 62.78
90° running turn	2.20 ± 0.40	240.28 ± 83.01	0.60 ± 0.17	20.80 ± 6.56	3.12 ± 0.88	253.13 ± 91.06	1.45 ± 0.73	61.94 ± 31.19
45° cutting maneuver	2.52 ± 0.50	284.58 ± 85.73	0.61 ± 0.23	43.97 ± 35.24	3.50 ± 1.29	310.16 ± 144.96	2.11 ± 1.38	120.90 ± 110.35
Mean	1.82 ± 0.79	180.27 ± 98.86	0.60 ± 0.09	45.57 ± 15.56	2.52 ± 1.07	190.42 ± 106.46	1.28 ± 0.57	74.58 ± 26.66

*Data are presented as mean ± standard deviations; KFM, knee flexion moment; KAM, knee adduction moment*.

**Table 3:** Percent differences (*%Diff*) of discrete load metrics (peak and impulse).

**Locomotion task**	**KFM**		**KAM**	
	**Peak**	**Impulse**	**Peak**	**Impulse**
	**%Diff**	**%Diff**	**%Diff**	**%Diff**
Walking straight	44.3 ± 70.8	27.4 ± 83.9	39.1 ± 101.0	62.0 ± 253.1
90° walking turn	47.1 ± 60.6	6.7 ± 31.3	82.4 ± 110.5	69.3 ± 127.5
Moderate running	24.7 ± 33.0	0.65 ± 37.2	68.7 ± 94.5	42.7 ± 108.9
Fast running	37.2 ± 68.7	6.8 ± 40.7	123.5 ± 124.1	94.2 ± 145.3
90° running turn	44.9 ± 45.2	12.1 ± 46.5	159.8 ± 157.1	230.0 ± 179.9
45° cutting maneuver	44.1 ± 60.7	10.0 ± 42.6	308.2 ± 356.5	470.0 ± 702.0
Mean	40.4 ± 56.5	10.6 ± 47.0	130.3 ± 157.3	161.4 ± 252.8

*Data are presented as mean ± standard deviations; KFM, knee flexion moment; KAM, knee adduction moment*.

### Model Comparison

The changes in estimation accuracy due to independent model building for KFM and KAM for each of the analyzed locomotion tasks is presented in Table 4. Independent model building resulted in a lower *r* value for both KFM and KAM in the majority (five out of six) of the analyzed locomotion tasks in comparison to the combined estimation model. Across all locomotion tasks, mean RMSE and mean rRMSE increased for KFM^*^ (RMSE = 0.15, rRMSE = 1.18) and KAM^*^ (mean RMSE = 0.13, rRMSE = 0.26) due to independent model building.

**Table 4:** Increase (+) or decrease (–) in estimation accuracy (*r*, Pearson's correlation coefficient; RMSE, root-mean-squared error; rRMSE, relative root-mean-squared error) due to independent model building in comparison to the combined model.

**Locomotion task**		KFM^*^			KAM^*^	
	***r***	**RMSE** **(Nm/kg)**	**rRMSE** ** (%)**	***r***	**RMSE** **(Nm/kg)**	**rRMSE** ** (%)**
Walking straight	0.03	0.00	0.50	−0.20	0.05	2.64
90° walking turn	−0.02	0.03	1.56	−0.08	0.07	0.09
Moderate running	−0.02	0.18	1.31	−0.10	0.09	−1.58
Fast running	−0.03	0.15	0.90	−0.04	0.20	1.87
90° running turn	−0.08	0.11	0.85	−0.14	0.16	−0.87
45° cutting maneuver	−0.07	0.44	1.94	0.26	0.22	−0.57
Mean	−0.03	0.15	1.18	−0.05	0.13	0.26

KFM^*^, knee flexion moment; KAM^*^, knee adduction moment.

## Discussion

This study was aimed to develop and train an ANN model to estimate KFM and KAM during various locomotion tasks based on data obtained by two wearable sensors. The mobile assessment of knee joint loading enlarges the scope of diagnostic methods and disease management of KOA, which could help to improve disease treatment strategies and minimizing the risk of non-functional overreaching (e.g., pain).

The results of the study show a higher estimation accuracy of the KFM compared to the KAM over most locomotion task. However, estimation accuracy highly varied between tasks for both the KFM and the KAM, especially with increasing intensity and movement velocity. Apart from walking straight, for all locomotion tasks, a distinct reduced level of agreement was found between the ANN-estimated outcomes and reference data for the KAM (mean *r* = 0.39 ± 0.32, mean rRMSE = 29.9% ± 8.1%) in comparison to the KFM (mean *r* = 0.74 ± 0.36, mean rRMSE = 20.8% ± 5.7%). Discrete load metrics highlighted lower %Diff of KFM impulses in comparison to KFM peaks in all locomotion tasks, whereas %*Diff* of KAM impulses were lower compared to KAM peaks only in three out of the six locomotion tasks.

### Estimation Accuracy Across Different Locomotion Tasks

In general, when comparing the estimation accuracy across the different locomotion tasks, predictive power was always better and %Diff was always less for KFM than for KAM. On average, strong correlations (*r* = 0.74) and rRMSE of 20.8% for KFM and moderate correlations (*r* = 0.39) with rRMSE of 29.9% for KAM were found. Nonetheless, distinct differences between KFM and KAM estimation values were evident across the locomotion tasks.

For KFM, highest correlations with the inverse dynamics calculations were found for moderate running (*r* = 0.85), which is reinforced by lowest %Diff for both the peak and impulse of the KFM. The lowest correlations and largest rRMSE were found for fast running (*r* = 0.65; rRMSE = 25.5%). Nevertheless, %Diff for KFM peaks and impulses during fast running were lower than for most of the other locomotion tasks, except for moderate running. Interestingly, the largest %Diff was found for walking straight, while %Diff of moment integrals were in general lower compared to %Diff of peak moments. These findings indicate that our ANN-configuration is more appropriate for estimating knee joint loading over the stance phase than for estimating the peak moment of the stance phase. In particular, during walking straight, the low knee flexion moment peaks and impulses might account for the strong correlations but large %Diff. Albeit, for KFM generally high agreement was found for ANN-estimated outcomes, with a reduced performance for the high intensity movements running and cutting maneuvers. In contrast, in these movements lower %Diff occurred to the lower-intensity movements.

For the estimation of KAM, overall weak to strong correlations were found for the analyzed movements. Estimation accuracy was highest in walking straight (*r* = 0.71, rRMSE = 22.3%). Mediocre correlations were found in moderate running as well as 90° walking/running turns (0.40 ≤ *r* ≤ 0.56), and low or negative correlations in fast running and 45° cutting maneuvers (−0.05 ≤ *r* ≤ 0.21). With regard to rRMSE, alterations of locomotion speed (walking to running) and direction (turning and cutting) led to slight reductions in accuracy of the ANN estimations. Concomitant, large increases in %Diff along with high variability were detected in fast running, 90° running turns and 45° cutting maneuvers (KAM impulse: 94.2, 230.0, and 470.0%, respectively). A potential reason for the less estimation accuracy and larger differences for movements with increased velocity and changes of direction might be the higher variation in the execution of these movements, while locomotor tasks such as walking or moderate running are performed automatically with repeatable characteristics (Schmidt and Lee, 2011). Similarly, variability in estimation accuracy was also shown by Fluit et al. (2014), evaluating a prediction model for GRFs and moments during various activities of daily living by means of 3D full-body motion.

However, a generalization of the estimation accuracies cannot be deduced, as a reduced estimation accuracy in continuous outcomes does not necessarily result in an inaccurate estimation of discrete variables, as it was seen in the KFM during fast running. Similarly, good agreement in continuous outcomes does not implicate accurate estimation of discrete load metrics, as seen in 90° running turn. Furthermore, it must be noted that most KFM and KAM show high standard deviations, which indicates a wide dispersion across participants. Nonetheless, %Diff of KFM were entirely lower in the impulses compared to the peak values. In contrast, %Diff of KAM impulse were lower compared to the peak values only in three out of the six locomotion tasks (90° walking turn, moderate and fast running). Summarized, KAM estimations were less accurate both for continuous and for discrete outcomes compared to KFM and should therefore be treated with caution. The more pronounced characteristic changes in the KAM time series between locomotion tasks in comparison to the KFM time series are a potential reason for the reduced estimation accuracy in KAM (see Figures 2, 3).

Furthermore, with respect to the comparison of a combined estimation model for KFM and KAM and independent models for KFM and KAM, the results show that an independent model building leads to slightly decreased estimation accuracy of the KFM and a more pronounced decrease of the KAM, concomitant with increased RMSE and rRMSE in the investigated locomotion tasks. Hence, if only one variable was chosen as an output, decreased performance for the model was observed, indicating that cross-dependencies between input and output in the combined estimation model clearly affected the estimation accuracy. Overall, the combined estimation model for KFM and KAM presented a fair estimation accuracy, especially, in the low-intensity movements.

### Comparison of Different Wearable Measurement Systems

A novel machine learning based method was developed and applied in this study to estimate KFM and KAM based on data obtained by two wearable sensors integrated in a knee sleeve. Various approaches have experienced progressive advances to assess the mechanical loading of KOA patients in their habitual environment over the past years. The majority of the approaches were based on analytical biomechanical models, which typically determine joint moments by means of the inverse dynamics calculations. As a consequence, GRF measurements and kinematic data are necessary to perform such analysis (Whittlesey and Robertson, 2014).

Van den Noort et al. presented in 2011 an instrumented force shoe as an alternative to force plate measurements. Subsequently, an ambulatory measurement system, consisting of the instrumented force shoe and an inertial measurement system combined with a linked-segment model, was used to compare KAM measures with a laboratory based system in KOA patients (van den Noort et al., 2013). Limited accuracy was shown and the authors concluded that a more advanced calibrated linked-segment model should be investigated (van den Noort et al., 2013). As an alternative to a direct measurement of GRF, Karatsidis et al. (2016) estimated GRF by means of a full-body inertial motion capture system during walking. Their results showed for the comparison with an optical motion capture system higher *r* values (range 0.82–0.99 and 0.76–0.99 for the inertial and optical motion capture systems, respectively) and lower rRMSE values (range from 5 to 15% for both systems) compared to the KFM and KAM estimations present in this study. More recent studies from Dorschky et al. (2019) and Konrath et al. (2019) used inertial motion capturing and musculoskeletal modeling to estimate biomechanical variables, such as joint kinematics and kinetics without GRF data. Dorschky et al. (2019) presented high correlations for sagittal plain kinematics (*r* > 0.93) and kinetics (r > 0.90) in gait and running. In accordance, Konrath et al. (2019) estimated the KAM and the tibio-femoral joint contact force during daily living activities (e.g., stair walking) with moderate to strong correlation coefficients. However, such approaches using inertial sensor data and musculoskeletal models require more IMUs (seven IMUs in Dorschky et al., 2019 and 17 IMUs in Konrath et al., 2019) compared to this study's approach.

Parallel to the analytical model development, an increasing number of machine learning approaches have been explored to simplify data acquisition and modeling strategies to estimate target variables, such as the KAM (Liu et al., 2009; Favre et al., 2012; Wouda et al., 2018). ANN modeling does not require modeling of the musculoskeletal system, as the relationship between the input IMU signals and the target variables is build up during the training process of the model (Halilaj et al., 2018; Wouda et al., 2018). However, ground truth reference data, such as the inverse dynamics-calculated KFMs and KAMs, are required during the supervised learning process of the model. Providing a large amount of known output data is essential to establish a robust model (Sivakumar et al., 2016; Halilaj et al., 2018). Wouda et al. (2018) used similar ANN modeling to the one used in this study for estimating vertical GRF and sagittal knee kinematics during running. The estimated vertical GRF profiles of their non-personalized ANN developed by eight participants showed a higher correlation (*r* > 0.90) to the actual force time series. The slightly reduced estimation accuracy in the current study (*r* < 0.85) may depend on the variety of locomotion tasks included in the model building. A more locomotion task-specific modeling may lead to an increased estimation accuracy for individual tasks, but has the disadvantage that each task must be modeled by itself (Wouda et al., 2018). In consequence, the combination with an activity recognition approach could help to select individual estimation models in practical applications.

### Limitations

Certain limitations of this study need to be considered when interpreting the results. One consideration worth noting is that the estimation accuracy depends on the neural network architecture. The ANN was built on previous work (Favre et al., 2012; Wouda et al., 2018), which highlighted that such configuration is capable of mapping non-linearity between input and output; however, other model specifications may result in an improved estimation accuracy. The ANN was trained with data from multiple participants as well as various locomotion tasks, which should rather lead to a less participant- and task-specific but a more generic model. As a consequence, this approach rather yields a decreased performance due to a lack of individualization, but has the advantage that not every new user needs to perform a training phase (Favre et al., 2012; Wouda et al., 2018). Further research is necessary to assess if a single participant learning approach increases the estimation accuracy. Another limitation is that we included a homogeneous group of participants consisting of only males without any musculoskeletal disorders and the translation of the results to the target group of KOA patients remains speculative. Nonetheless, future clinical studies may benefit from the use of the method developed in this study, especially in low-intensity movements (Richards et al., 2017). Beyond, the sample size was rather small, including 13 participants. Similar investigations included comparable numbers of participants (e.g., sample of eight participants in Wouda et al., 2018 or sample of 17 participants in Leporace et al., 2015). The small sample size potentially limits the outcome, as the robustness of the relationship between the input and output variables of the ANN depends on the amount of training data (Sivakumar et al., 2016; Ancillao et al., 2018; Halilaj et al., 2018). Finally, it cannot be fully ensured that the fixation technique excluded any oscillations or misalignment of the IMUs, even though the exact fit of the sleeve and the sensors was repetitively checked. However, the wearable sensors were integrated in a knee sleeve on purpose to mimic natural effects and to capture IMU signals closely related to the joint under investigation.

## Conclusion

This study demonstrated the potential of estimating KFM and KAM for various locomotion tasks using a minimal body-worn sensor setup consisting of two IMUs integrated in a knee sleeve. The agreement between the ANN-estimated outcomes and inverse dynamics-calculated data was strong for the majority of analyzed locomotion tasks in the KFM and moderate in the KAM. Overall, higher estimation accuracies were seen for the KFM in comparison to the KAM across all locomotion tasks. The accuracy limitations especially of KAM estimation makes prediction of knee joint loading challenging. In order to reach an acceptable level of accuracy related to critical changes due to KOA, typically characterized by relatively small kinetic differences, a participant- or task-specific modeling could be helpful. This has important implications for the development of wearable devices as well as for scientific research on KOA. The highest estimation accuracy for both KFM and KAM of walking straight matches the main characteristic of KOA therapy and treatment by low-intensity movements (e.g., walking). Looking ahead, wearable technology could serve as a rehabilitation aid for patients with KOA leading to an improved load management, which could result in a slower progression.

## Data Availability Statement

The datasets generated for this study are available on request to the corresponding author.

## Ethics Statement

The studies involving human participants were reviewed and approved by ethics committee of the Karlsruhe Institute of Technology. The patients/participants provided their written informed consent to participate in this study.

## Author Contributions

BS, FK, SR, TS, and SS were involved in the design of the study. BS, FK, and SR carried out all data collection and analysis. BS, FK, SR, and TS were involved in the interpretation and discussion of the results. BS took the lead in writing the manuscript. All authors provided critical feedback and contributed to the final manuscript.

### Conflict of Interest

The authors declare that the research was conducted in the absence of any commercial or financial relationships that could be construed as a potential conflict of interest.
